# The changing role of a Vaidya (non-codified traditional doctor) in the community health of Kerala, Southern India: comparison of treatment-seeking behaviours between the Vaidya’s patients and community members

**DOI:** 10.1186/s13002-015-0042-2

**Published:** 2015-07-10

**Authors:** Sachi Matsuoka

**Affiliations:** Graduate School of Asian and African Area Studies, Kyoto University, Room #AA439, Research Bldg. No. 2, Yoshida-Honmachi, Sakyo-ku, Kyoto 606-8501 Japan

**Keywords:** Vaidya, Non-codified traditional doctor, Kerala, South India, Medical pluralism, Traditional medicine, Treatment-seeking behaviours

## Abstract

**Background:**

This study aimed at exploring the roles of a Vaidya – an uncodified traditional doctor – in a community in Kerala State, India. Special attention was paid to the characteristics of the Vaidya’s patients in comparison with the treatment-seeking behaviour of the community members.

**Methods:**

Both qualitative and quantitative data about the Vaidya, 97 of his patients, and 31 community members were gathered via participatory fieldwork and open-ended interviews.

**Results:**

It was found that the community members seldom consulted the Vaidya who lived in their community; thus, the role of the Vaidya as the community’s primary health care provider had nearly disappeared. Nonetheless, the Vaidya was deeply respected as one of the community’s leaders by its members because of the spiritual and financial support he provided to them. On the other hand, a number of patients visited the Vaidya from outside the village, which implied that the Vaidya played a new role under the changes caused by medical pluralism. Even a codified traditional medicine, Ayurveda, was less popular among the community members. These findings were interesting, because while the traditional Indian medical system has been becoming popular and common in other societies, such as European societies, as an alternative medicine, the traditional medical system was becoming less important in the rural Indian context.

**Conclusion:**

It is thus concluded that the medical practice has changed depending on its cultural and social contexts, even though its medicinal effects had been proven by scientific survey.

## Background

It has been conventionally recognized that most people still used traditional medical systems, such as Ayurveda, for primary care in rural India regardless of the spread of western biomedicine, called ‘allopathy’ [9]. In reality, the Indian government has been recognizing the value of traditional medical systems, such as Ayurveda, Yoga, and a localized ‘homeopathy’ which originated in Europe but differs from biomedicine, and expending effort to integrate them into the official education of the modern medical system, to some extent [12]. This medical pluralism of the community health system has been an important study subject as researchers seek to understand treatment-seeking behaviour and propose appropriate health policy [2, 8].

While Ayurveda, Siddha, Unani, homeopathy, and allopathy have all been registered as ‘codified’ medical systems, there are also ‘non-codified’ medical systems. In South India, non-codified medical practitioners, called Vaidyas, have been recognized as playing central roles in community health in rural villages [16]. However, recent studies reported that the Vaidyas’ roles were decreasing because (1) codified medical systems were now available in many of rural villages, (2) rural people preferred to use allopathy rather than the Vaidya, and (3) Young people were not interested in learning non-codified medical systems, therefore there were less young Vaidyas [6, 7, 15, 16]. These previous studies suggested a conflict because the two different types of traditional medical systems, that is, codified and non-codified, showed opposite patterns; while the people still like to use the ‘codified’ traditional medical system, they were less likely to use a ‘non-codified’ one. While previous studies have been conducted from descriptive medical anthropological viewpoints or have been public health studies based on administrative data at the macro level, no studies have disclosed the current roles of the Vaidyas under these social and medical changes using qualitative methods at the community level.

This study therefore aimed at exploring the role of the Vaidya – an uncodified traditional doctor – in a community in Kerala State, India. Special attention was paid to the characteristics of the Vaidya’s patients in comparison to the treatment-seeking behaviour of the community’s members. The findings will be useful for constructing appropriate medical health systems in India’s medically pluralistic communities.

## Methods

### Study site

Kerala is located in the southwestern part of India covering the Malabar Coast and is a small state with a land area of 38,863 km^2^ (only 1.18 % of the total land area of the country). Geographically speaking, the state can be divided into a hilly area, a semi-hilly area, and plains with varied vegetation types along the line of the Western Ghats. Historically speaking, Kerala had been a prominent spice exporter since 3,000 BC, so not only Hinduism developed in this region; Muslim traders and Christian European traders (14th Century) continuously influenced the area. Accordingly, although Hinduism is practiced by the majority (56 %) of the population, Muslims and Christians account for 24 % and 19 %, respectively [11].

Kerala has the highest Human Development Index (HDI) in the country at 0.790 (Human Development Report 2011), even though the state’s per capita income (80,924 Indian rupees [5]) in 2011–12 is lower than the average for developing countries. This human development experience has been called the ‘Kerala Model’ in the Human Development Report authorized by the United Nations Development Program (UNDP). In this model, an increased number of highly educated people and a prolonged life expectancy have been noticed. However, in reality, the unemployment level was high due to slow industrial development, regardless of the high education level. A rapid increase in the society’s average age was also recognized as a new problem.

This study was conducted in a coastal village in the Eranakulam District, Kerala. The village is located approximately 30 km from Cochin City, the biggest commercial centre, so the village has been recognized as suburban and susceptible to various kinds of modernization. About half of the villagers have been engaged in either fishery or fish processing industries [3]. Many of those villagers were so poor that they were provided with assistance in the form of essential foods (e.g., rice, sugar, and vegetables) by the local government; females received work assistance by being engaged as governmental labour to satisfy their need for minimum necessary income. The proportion of people in scheduled castes (15.9 %) was higher in the village than the mean in other parts of the district (8 %) [3]. In addition to Hindu temples, there were a Christian church and mosques, displaying religious diversity.

There were one official community health centre, one private allopathy hospital, a number of allopathy pharmacies, one homeopathy clinic, homeopathy pharmacies, Ayurvedic pharmacies, and one Vaidya in the village.

### Fieldwork

Fieldwork was conducted for a total of 90 days in 2012 and 2013. The author stayed in India for a total of 154 days, from 12 August to 14 October 2012, from 14 January to 5 Match 2013, and from 24 August to 4 October 2013, to conduct this study. The Vaidya and all other participants agreed to participate in this study after they were informed of its nature, what their participation would entail, and their right to withdraw during any stage of the research without having to give reasons for doing so. The privacy policy was also explained to all participants, which dictates that all personal information collected in this research must be kept anonymous and that the results will be published in academic journals or presented at meetings. Following this, fully informed oral consent was obtained from all participants before proceeding with this study. This study was approved by the Kyoto University Graduate School and the Faculty of Medicine Ethics Committee on 7 August, 2013 (approval number: E1824).

#### Observation and interview with the Vaidya

The Vaidya in the study village was chosen as a key informant because of the following three reasons. First, the study village, where various kinds of medical systems existed, represented a medically pluralistic society, and the Vaidya was the only non-codified practitioner. Second, the study village was economically poor but geographically near the commercial centre, displaying a representation of the current situation of those Indian villages which were experiencing rapid socioeconomic changes. Therefore, the Vaidya encapsulated the situation of non-codified practitioners today. Third, in the author’s pre-survey, it was confirmed that a high enough number of patients (approximately 5–10 per day) visited the Vaidya, indicating their confidence in his ability.

Both qualitative and quantitative data ware gathered via participatory fieldwork and open-ended interviews from 15 September to 5 October 2012. Participatory observation and an interview with the Vaidya were conducted at his place of practice, called ‘*ashram*’; note that the term *ashram* originally referred to Hindu hermitage. During the survey, the author, who was educated as a pharmacist in Japan, was mainly stationed at the pharmacy section in the Vaidya clinic and observed closely the prescription, processing, and dispensing of medicine through participatory observation. During this period, the author informally interviewed the Vaidya on various items, such from his life history, philosophy of treatment, medical services, treatment methods based on plants and animal substances, treatment based on religious belief, and treatment using commercially manufactured medicines. The Vaidya did not hesitate to answer any questions and wished to be open about his medical practices and formulations (i.e., ‘recipe’ of medicine) to the public, insisting that the efficacy of his medicine should contribute to others.

#### Interview of the Vaidya’s patients

In order to understand the characteristics of the Vaidya’s patients, the author alone conducted face-to-face interviews with structured questionnaires at the clinic, while the patients were waiting for medicines, from 1–28 February 2013. All of the patients, 97 in number (64 males; 33 females), who visited the Vaidya during the research period agreed to participate in the survey. Since all but one of these patients came from various areas other than Kerala and were educated enough to be able to speak English – one of the official languages of India – English was used to interview them. The local Malayalam language was used to interview the one local village patient.

The questionnaire included the following items: (1) social-demographic data (name, sex, age, occupation, religious belief, place of residence), (2) reason for choosing the Vaidya for treatment, and (3) current symptoms/complaints, previous treatments for them, and medical history. In the case that the participants’ symptoms were too severe to answer all of the questions, the attendant family member was asked to answer in their stead.

Based on the patients’ reports and diagnoses by the Vaidya, the author, who is a pharmacist well trained to provide care in developing countries, classified the patients’ symptoms into International Classification of Diseases-10 (ICD-10) categories for analytical convenience. The levels of seriousness were also classified into the categories of ‘severe’, ‘moderate’, and ‘light’; this classification was made based on the type of disease, influence on activity of daily living (ADL), and estimated life prognosis.

#### Interview of community members

In order to understand the treatment-seeking behaviours of the community members in comparison to the Vaidya’s patients, face-to-face interviews with structured questionnaires were conducted in the village by the author, mainly in their local language of Malayalam, by means of visiting the respondents’ houses from 24 August through 3 October 2013; in a few cases, English terms were used to describe the names of diseases and symptoms. The community members, 31 in number (22 males and 9 females), who have recent medical histories of diseases/illnesses (*reagam* in Malayalam) and live within 1.5 km of the Vaidya clinic in Kerala were selected as follows. The author asked two public health nurses about community members who have/had *reagam* and visited most of the households, excepting those that were absent, in the area to find potential informants who matched the criteria. The concept of *reagam* includes unhealthy status both mentally and physically, but excludes external wounds. The 31 community members were selected and agreed to participate in this survey. The population of the village was 35,906 [3]. Although no detailed demographic data was available, the study site (i.e., a 1.5 km radius around the Vaidya) covered approximately 5 % of the village’s population.

The respondents were asked about (1) social-demographic data (name, sex, age, literacy, education, religion, occupation of householder, economic status) and (2) current complaints, previous treatments, and medical history. Subjective scaling of economic satisfaction and quality of life were also requested and to be published elsewhere. The interviews were extended further as open-ended discussions to study medical belief, their faith, and their socially oriented societies.

## Results

### Features of the Vaidya, traditional medical practitioner

The Vaidya chosen for this study was a 50-year old (born in 1964) male who was born in the study village. He ran the clinic with his mother, his older sister, and six assistants. He was not from a medical family pedigree, but a Hindu family. In his early twenties, he started to practice yoga, and he was awakened to the spiritual way of living. After that, he followed various masters/saints (*guru*) to learn Ayurveda, Siddha, traditional martial arts (*kalarippayattu*) in and outside Kerala, and also sometimes lived with ethnic minority people in the West Ghats mountain area to learn about their indigenous medical system. He returned to his native village and launched a clinic in 2000 after he was recognized as a *swami* – a Hindu priest – due to his training experience. Accordingly, his clinic was also called an *ashram* – the name for a Hindu hermitage where a Hindu holy person lives.

In response to open-ended questions, the Vaidya insisted that he has originally integrated several kinds of medical systems into one system. The system he provided was based mainly on Ayurveda theory, while he also applied a number of medical systems he learned. He prepared over 50 kinds of herbal medicines in the clinic. Half of these were prepared from fresh and/or dried medicinal plants or animal substances which he purchased in markets in urban areas; the rest were Ayurvedic/Siddha pharmaceutical companies’ products. The medicinal plants are collected/grown mainly in the semi-mountainous area and sold in the urban market.

He did not fix his consultation day and usually admitted between one and three inpatients while examining five to ten outpatients per day. Although he did not require payment for his medical practices, except for medicine provisions, free-will donations were moderately encouraged. The cost of medicine ranged from 700 to 5,000 Indian rupees, though it depended on the type of disease/illness and the length of the medication period. The medicine cost was recognized as very expensive. The Vaidya, therefore, professed that he did not require any payment from those who were not able to afford the cost and suffered from diseases/illness. However, there were no patients who asked the Vaidya to exempt the payment during this study term. It is noteworthy that during the author’s participatory observation, the Vaidya made large financial contributions to the village community (e.g., donation for the village ceremony and to the public sector).

### Characteristics and symptoms of the Vaidya’s patients

During the study period, 97 patients visited and received treatment from the Vaidya; among them, 66.0 % (64) and 34.0 % (33) were males and female, respectively, and the average age was 41.4 years (standard deviation [SD] = 16.7) (Table 1). Table 1 also shows the comparison of the patients’ characteristics with those of the residents of Kerala overall. As a result, the patients were characterized by high proportions of males (67 % vs. 47.97 % in the state), of Hindus (77.3 % vs. 56.2 % in the state), of visitors from outside the village (99.0 %), of English speakers (99.0 %), and of professional occupation (36 %). In terms of gender, it is noteworthy that 66.6 % (50 patients) of Hindus (75), 81.8 % (9) of Muslims (11) and 54.5 % (6) of Christians (11) were males. It is likely that the Muslim and some Hindu females were especially hesitant to consult the Vaidya, who is a male. It is apparent that the Vaidya is preferred by Muslim males and many Hindu males, but not by Christians due to their religious beliefs. Unexpectedly, there was only one patient from the same village as the Vaidya. Although not shown in Table 1, it is noteworthy that the patients included one biomedical (allopathy) doctor and two nurses.

**Table 1:** Characteristics of the study participants and all Kerala people

		Vaidya patients^a^ (*N* = 97)	Village patients^b^ (*N* = 31)	All Kerala^c^ (*N* = 33,406,061)	Comparison Vaidya vs All Kerala
Sex	Male	67 %	71 %	47.97 %	*χ* ^2^ = 14.5
	Female	33 %	29 %	52.02 %	*P* <0.0001
Age		41 (SD 16.7)	48 (SD 21.1)	32	*t* = 8.11
					*P* >0.05
Religion	Hindus	77.3 %	71 %	56.2 %	*χ*2 = 23
	Christians	11.3 %	26 %	19.0 %	*P* <0.0001
	Muslims	9.3 %	3 %	12.0 %	
	Others	0 %	0 %	12.8 %	
Place of living	In the village	1 %	100 %	NA	
	Other villages in Ernakulam (inc. Cochin city)	57 %	0 %	NA	
	Other district in Kerala	39 %	0 %	NA	
	Other states	2 %	0 %	NA	
	Over seas	1 %	0 %	NA	
Occupation	Professional/skilled worker	36 %	0 %		
	Manual labor	19 %	26 %		
	Agriculture/fishery	3 %	13 %		
	Homemaker/Student/Retired	38 %	61 %		
	Unknown	3 %	0 %		

Table 2 shows the symptoms and seriousness of illness of the patients visiting the Vaidya. In this interview, only two patients explained their complaints based on Ayurvedic theory, while the remaining used the English name of the diseases in Western biomedicine. Some patients brought the result papers from medical check-ups at a private diagnostic service in a town. Severe cases (23.7 % in total), such as malignant neoplasm, circulatory diseases, comatose state due to allopathy operation failure, and advanced Parkinson’s disease, were observed. Moderate cases (40.2 % in total) were represented by chronic diseases: articular rheumatism (‘diseases of the musculoskeletal system and connective tissue’ in Table 2), diabetes, nerve pain, and thyroid deficiency. Light cases (36.0 % in total) included symptom of wounds, common cold, loss of hair, declined eye sight, slight back pain, scars from insect bites, and others.

**Table 2:** Classification and severity of diseases among the patients visiting the Vaidya

Seriousness	ICD-10 classification No. disease	Total	Proportion
Severe 23.7 %(23)	2 Neoplasms	7	7.2 %
	6 Diseases of the nervous system	5	5.2 %
	9 Diseases of the circulatory system	4	4.1 %
	11 Diseases of the digestive system	3	3.1 %
	4 Endocrine, nutritional and metabolic diseases	2	2.1 %
	1 Certain infectious and parasitic diseases	1	1.0 %
	19 Injury, poisoning and certain other consequences of external causes	1	1.0 %
Moderate 40.2 %(39)	13 Diseases of the musculoskeletal system and connective tissue	19	19.6 %
	4 Endocrine, nutritional and metabolic diseases	6	6.2 %
	6 Diseases of the nervous system	4	4.1 %
	5 Mental and behavioral disorders	3	3.1 %
	10 Diseases of the respiratory system	3	3.1 %
	11 Diseases of the digestive system	3	3.1 %
	12 Diseases of the skin and subcutaneous tissue	1	1.0 %
Light 36.0 %(35)	12 Diseases of the skin and subcutaneous tissue	12	12.4 %
	10 Diseases of the respiratory system	6	6.2 %
	13 Diseases of the musculoskeletal system and connective tissue	6	6.2 %
	21 Factors influencing health status and contact with health services	4	4.1 %
	11 Diseases of the digestive system	3	3.1 %
	1 Certain infectious and parasitic diseases	1	1.0 %
	3 Diseases of the blood and blood-forming organs and certain disorders involving the immune mechanism	1	1.0 %
	4 Endocrine, nutritional and metabolic diseases	1	1.0 %
	7 Diseases of the eye and adnexa	1	1.0 %
Grand Total		97	100 %

Table 3 shows the patients’ treatment-seeking behaviours before visiting the Vaidya, broken down by their religion and the severity of their ailment. Among the patients, 45.3 % (44) had consulted other medical institutes before visiting the Vaidya. In cases classified as severe, a high proportion of the patients had been treated with other types of medical systems, especially allopathy, before visiting the Vaidya: 14 out of 16 Hindus and 5 out of 6 Muslims. In moderate, 15 out of 29 Hindus, 2 out of 6 Christians, and 3 out of 4 Muslims had used other medical systems, especially allopathy, as a first choice. Of all the categories, Hindus with light severity displayed the highest proportion of choosing the Vaidya as their first treatment (27 out of 30 patients, or 90.0 %). While most of the Hindu or Christian patients were ‘moderate’ or ‘light’, the majority of the Muslim patients were ‘severe’. Although a high proportion of Christian patients chose the Vaidya for their first treatment, the treatments were adopted for moderate or light cases only.

**Table 3:** Treatments consulted before the Vaidya, broken down by religion and seriousness

				Previous treatments				Seriousnes vs treatment done before Vaidya or not
		None	Allopathy	Homeopathy	Ayurveda	Traditional medicine	Total	
Hindus								*P* <0.0001
	Severe	2	13			1	16	
	Moderate	14	12		2	1	29	
	Light	27	3				30	
	Subtotal	43	28		2	2	75	
Christians								*P* >0.0001
	Severe	0		1			1	
	Moderate	4	1			1	6	
	Light	3	1				4	
	Subtotal	7	2	1	0	1	11	
Muslim								*P* >0.0001
	Severe	1	4			1	6	
	Moderate	1	3				4	
	Light	1					1	
	Subtotal	3	7	0	0	1	11	
Total		53	37	1	2	4	97	

### Community members’ treatment-seeking behaviours

During the study period, 31 community members were interviewed; 71.0 % (22) and 29.0 % (9) were males and females, respectively, with a mean age of 48.2 years (SD = 21.14) (Table 1). As shown in Table 1, Hindus, Christians, and Muslims accounted for 71.0 % (22), 25.8 % (8), and 3.2 % (1), respectively. In addition, 51.6 % (16) of the respondents’ household were engaged in fishery, and 22.6 % (7) were manual labourers, such as carpenters and painters.

Among the respondents, while 67.7 % (21) used one type of medical system for their illness, the others (32.3 %) used two types of medical systems. Table 4 shows the diseases and treatments for them. Since cases were counted as both when two types of medical systems were used for one disease, the total number of treatments was 41, higher than the number of patients. Allopathy was used the most frequently (77.4 %), followed by homeopathy (32.3 %) and Ayurveda (12.9 %). Folk treatments which used medicinal plants were observed in three cases, and all of these cases were treated at home without consulting any non-codified practitioners. Although not shown in Table 4, allopathy was the most common treatment (71.4 %), even among those who used only one treatment. Combination use of allopathy and homeopathy was the most common (70.0 %) among those who used two types of medical systems.

**Table 4:** Deseases and treatments for them among the village inhabitants

			Treatment^a^		
Disease	Allopathy	Homeopathy	Ayurveda	Medical plants	No. of patients
1 Infectious and parasitic diseases	1				1
4 Endocrine, nutritional and metabolic diseases	9	2			9
5Mental and behavioral disorders	2	2			3
6 Diseases of the nervous system			1		1
7 Diseases of the eye and adnexa	2				2
9 Diseases of the circulatory system	2	1			2
10 Diseases of the respiratory system	4	2		1	5
11 Diseases of the digestive system	1		1		1
13 Diseases of the musculoskeletal system and connective tissue	2	2	2	2	5
14 Diseases of the genitourinary system	1	1			2
Total	24	10	4	3	31

^a^Since the case was counted for both when two types of medicines were used for one illness, the total number of treatments was 41, but not 31

Endocrine, nutritional, and metabolic diseases, such as diabetes and thyroid disorders, were treated at least once with allopathy; two such cases were treated in combination with homeopathy. Both nervous system disorders and diseases of the musculoskeletal system and connective tissues (e.g., rheumatics) were more likely to be treated with a non-allopathy medical system than other diseases.

Table 5 shows these non-allopathy treatments broken down by the patient’s religion and the illness’s severity. Hindus used allopathy in 17 out of 22 cases. Christians used allopathy for all cases with or without using other types of medical systems and regardless of the severity. Throughout all cases, the use of Ayurveda was rare. Several informants insisted that Ayurveda required time to achieve a cure or a remission, while the recovery process was obvious when cured with a biomedical treatment.

**Table 5:** Treatment of the village inhabitants, broken down by the religion and the severity. In case that two or more treatments were used for one illness, the case was counted for all treatments used

	Treatment		Allopathy	Homeopathy	Ayurveda	Medicinal Plants	No. of Patients
Hindus		Severe	1		1		1
		Moderate	12	4	1		15
		Light	4	3	1		6
		Subtotal	17	7	3		22
Christians							
		Severe	1	1	1	1	2
		Moderate	5	2		1	5
		Light				1	1
		Subtotal	6	3	1	3	6
Muslims							
		Light	1				1
		Subtotal	1	0	0	0	1
	Total		24	10	4	3	31

## Discussion

Previous studies have reported the conflicting conclusions that the codified traditional medical systems were still very common in community health care while the non-codified ones were at risk of disappearing [6, 7, 16]. In this regard, this study found that the community members seldom consulted the Vaidya who lived in their community; thus, the roles of the Vaidya as the community’s primary health care provider almost disappeared. On the other hand, interestingly, a number of patients visited the Vaidya from outside the village, which implicated that the Vaidya played a new role in the medical pluralism resulting from these changes. Please note that some limitations exist in this study. First, since the population of the village is 35,906 (2013), the studied area in the village had to revolve around the Vaidya’s clinic. This potentially limits the generalizability of the results to other areas in India. Second, because only community members who met certain criteria and agreed to this study participated on a voluntary basis, the number of participants is limited, causing a selection bias. Third, there was not a survey of people living in the urban area conducted for comparison to the community members. This limits the interpretation of health-seeking behaviours in general Kerala.

The first part of the discussion focuses on the reason for the disappearance of the Vaidya’s role in the community’s health care. The findings of this study suggested that Ayurveda itself was less frequently used, even though it was codified. Ayurveda was chosen for some specific diseases which the community members recognized were not entirely cured by allopathy. In answering questions about the reasons they did not use the Vaidya and the low frequency of Ayurveda use, several informants insisted that such medical systems required long-term lifestyle modifications (e.g., diet, activity, and religious matters) and thus took time to achieve a cure or a remission, while the recovery process was obvious when cured with a biomedical treatment. Allopathy was consequently more believed to cure their illness quickly than Ayurveda. For the local community members, illnesses were not consciously separated from diseases, so when illnesses disappeared, diseases seemed to disappear at the same time. Open-ended interviews revealed that a difference between Vaidya patients and local members is the awareness of the difference between illnesses and diseases.

The cause of illness has been considered a factor in treatment-seeking behaviours. For example, previous studies suggested that illness caused by an ‘area-characteristics etiology’ (e.g., necromantic sorcerer) was traditionally treated by ethno-medicine in principle [1, 10, 14]. In other cases, illness with an area-characteristics etiology was still treated by ethno-medicine, but other illnesses, such as external injury, severe diseases, and communicable diseases, were preferably treated with modern medicines at the early stages of development [4, 13]. In this study site in India, the cause of illness was mostly recognized in a modern medical context, even by local community members.

Regarding treatment by the Vaidya, the cost is much higher than that of the codified medical systems, including traditional ones; this was partly caused by public financial assistance only being available for codified allopathy, homeopathy, Ayurveda, and Siddha, but not for un-codified medical systems. The community members were able to consult biomedical health services free of cost in the village. The Vaidya insisted that he never asked the economically poor patients to donate or pay for treatment or medicine. The village informants also agreed that the Vaidya might not ask for payment. However, no patients were observed asking the Vaidya to exempt them from payment during the study term. This fact shows that the cost of treatment is not a reason for community members to avoid the Vaidya. The community members do not rate handmade medicines particularly high since their life is full of handmade items (e.g. preserved food). On the other hand, some of the Vaidya’s patient informants insisted that handmade medicine is more effective than industrial products because they grow accustomed to purchasing expendable daily-life supplies that they do not make themselves. In addition, some of the Vaidya’s patient informants insisted that they liked to consult the Vaidya to feel a connection to nature while they lived a modernized life in a city far from it. They said they liked the traditional way of treatment for the same reason. Such preference for traditions and nature were thought to be linked to the herbal medicines and Ayurveda practices of the Vaidya. This tendency seemed to be strong among the highly educated patients.

This contrasted with the village inhabitant informants, who said that they preferred to use allopathy and industrially manufactured medicine. This does not mean that the community members neglect the Vaidya’s role in the community. The community members usually respect the Vaidya as one of the customary community leaders. The decrease of people’s dependence on the Vaidya and other traditional medicine is a nationwide tendency. Because of the spread of effective Western biomedicine and its improved availability (e.g. cost), Indian people prefer to use biomedicine. Therefore, it is natural that the inhabitants of the study village seldom consulted the Vaidya. Note that only a very small portion of urban people usually consult a Vaidya [16]. Therefore, if the author were to conduct similar research in the city, the use of a Vaidya may only be at an ignorable level; however, given the mass population of the city, the number of patients depending on the Vaidya is apparently large at each Vaidya’s station, as shown in this study.

The next part of the discussion is on the Vaidya’s role today. A previous study reported that 51 % of non-codified doctors attend up to five patients per week, whereas only 32 % of practitioners treat more than ten patients per week [16]. However, the Vaidya in this study attended five to ten patients each day, suggesting that a relatively large number of patients believed his treatment played an important role. The Hindus frequently used the Vaidya for treatment of severe to light symptoms. The reason why all cases of Muslims and the severe cases of Hindus mainly selected him as the second or third course of treatment in cases of severe or chronic diseases is thought to be that the Vaidya was an option to be used when allopathy or other types of medical systems failed to cure the illness. In the case of Hindus with light cases, the Vaidya was most often used as the first option. It is reasonable to assume that the Hindus trust and depend on the Vaidya because his title sometimes gives him recognition as a disciplinant of the Hindu, not just as a medical doctor. It is assumed that Hindus recognized that the treatment was effective for light cases; however, allopathy is more effective at treating severe cases. They are assumed to believe that the cause of illness is apart from spiritual/local etiology.

## Conclusion

This study focused on a Vaidya in order to examine the impact of medical modernization in Kerala by comparing people’s treatment seeking behaviours in urban areas to those in rural areas.

This study revealed that the Vaidya, who has been recognized as playing a key role in community primary health care, was not depended upon by the community’s members, but by highly educated urban people as a complementary and alternative medical system. The role of the Vaidya in rural areas has changed due to modernization.

These findings were interesting because while the traditional Indian medical system has been becoming popular and common in other societies (e.g., the Europeans) as an alternative medical system, it was becoming less important in the context of India. The previous studies showed that non-codified systems in particular are at the crossroad of extinction. The findings of this study suggest that social contexts, such as religious belief, an aspect of social context, directly affected treatment-seeking behaviours in modernized society where western etiologies are well known.

It is thus concluded that medical practice has changed depending on its cultural and social context, despite medicinal effects having been proven effective by scientific survey. Community health care systems have to be considered in multiple contexts, including social, economic, and environmental ones.
